# Red-Emitting Hybrid Based on Eu^3+^-dbm Complex Anchored on Silica Nanoparticles Surface by Carboxylic Acid for Biomarker Application

**DOI:** 10.3390/ma13235494

**Published:** 2020-12-02

**Authors:** João A. O. Santos, Alessandra M. G. Mutti, Airton G. Bispo-Jr, Ana M. Pires, Sergio A. M. Lima

**Affiliations:** 1 School of Technology and Sciences, São Paulo State University (Unesp), Presidente Prudente, SP 19060-900, Brazil; joao.antonio@unesp.br (J.A.O.S.); alegarbosamutti@gmail.com (A.M.G.M.); ana.maria@unesp.br (A.M.P.); 2 Institute of Biosciences, Humanities and Exact Sciences, São Paulo State University (Unesp), São José do Rio Preto, SP 15054-000, Brazil; 3 Institute of Chemistry, São Paulo State University (Unesp), Araraquara, SP 14800-900, Brazil; airton.germano.junior@gmail.com

**Keywords:** luminescent material, functionalization, lanthanide, β–diketone, SiO_2_, bioimaging

## Abstract

Luminescent organic-inorganic hybrids containing lanthanides (Ln^3+^) have been prominent for applications such as luminescent bio-probes in biological assays. In this sense, a luminescent hybrid based on dense silica (SiO_2_) nanospheres decorated with Eu^3+^ β–diketonate complexes using dibenzoylmethane (Hdbm) as a luminescent antenna was developed by using a hierarchical organization in four steps: (i) anchoring of 3-aminopropyltriethoxysilane (APTES) organosilane on the SiO_2_ surface, (ii) formation of a carboxylic acid ligand, (iii) coordination of Eu^3+^ to the carboxylate groups and (iv) coordination of dbm^−^ to Eu^3+^. The hybrid structure was elucidated through the correlation of thermogravimetry, silicon nuclear magnetic resonance and photoluminescence. Results indicate that the carboxylic acid-Eu^3+^-dbm hybrid was formed on the surface of the particles with no detectable changes on their size or shape after all the four steps (average size of 32 ± 7 nm). A surface charge of −27.8 mV was achieved for the hybrid, assuring a stable suspension in aqueous media. The Eu^3+^ complex provides intense red luminescence, characteristic of Eu^3+^ ^5^D_0_→^7^F_J_ electronic transitions, with an intrinsic emission quantum yield of 38%, even in an aqueous suspension. Therefore, the correlation of luminescence, structure, particle morphology and fluorescence microscopy images make the hybrid promising for application in bioimaging.

## 1. Introduction

Clinical diagnosis by imaging is expanding due to the development of sensitive and non-invasive techniques such as confocal or fluorescence microscopy, appearing as powerful tools for exploratory analyses of several biological processes and internal structural information of healthy and cancerogenous cells [1,2,3]. Among several biomaterials applied in bioimaging, luminescent biomarkers are widely used as contrast agents for in vivo and in vitro, clinical assays, acting as bio-probes responsible by imaging and clinical information of the cellular environment [4,5]. Therefore, new strategies towards the development and improvement of the luminescent and structural features of such bio-probes are of large social relevance.

Luminescent biomarkers reported in the literature can be classified in different categories, that is, organic dyes and fluorescent proteins [6], quantum dots [7], metallic nanoparticles [8], carbon dots [9] and Ln^3+^ or metallic ions in complexes or in inorganic hosts [10,11,12]. Each of those materials features chemical, physical and biological peculiarities that bring several advantages and disadvantages for use in cellular imaging [13].

In this sense, Eu^3+^ β-diketonate complexes excel for bioimaging [2,5,12,14] due to several features such as: (i) efficient antenna effect played by β-diketones to sensitize the Eu^3+^ luminescence, rendering biomarkers displaying relatively-high intrinsic emission quantum yield, (ii) excitation bands shifted to lower energy regions (near-UV or blue), (iii) large pseudo-Stokes shift, (iv) narrow Eu^3+^ emission bands within the red spectral region that arise from intraconfigurational *f–f* electronic transitions, leading to bright and pure red light emission, (v) emission within the biological window where light is less scattered and absorbed and easily differentiate from the biological autofluorescence, typically in the blue-green region [2] and (vi) long emission lifetime, within the 10^−6^–10^−3^ s range, favoring time-resolved luminescence methods to time-differentiate the cell autofluorescence, whose luminescence lifetime is typically shorter [15]. On the other hand, some drawbacks such as poor photostability upon UV excitation and low solubility in water need to be addressed for biological assays [16].

**Scheme 1:**
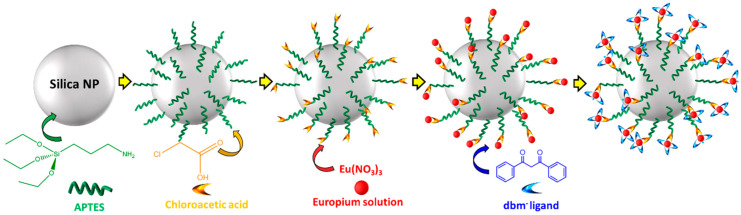
Scheme illustrating the sequence of reaction steps for the luminescent hybrid production.

Such shortcomings have been minimized over the immobilization of complexes in inorganic nanoparticles, for example, silica (SiO_2_) [12,17,18], clays [19], oxides [20], metals [21] or core-shell systems [22], making of these hybrid materials processed as stable colloidal suspensions displaying desirable luminescence and improved photostability. Moreover, the immobilization of the complex on the surface allows the nanoparticle internalization by cells, introducing a considerable number of luminescent centers within them, decreasing the dosage necessary to get first-rate clinical images. Among several support materials for the luminescent complex immobilization, SiO_2_ stands out as a biocompatible and non-toxic material for most cell lines due to the ease surface modification, entailing versatility for biofunctionalization by a substantial number of molecules [23,24,25]. Several methodologies can be employed to modify dense or mesoporous SiO_2_ surface to fabricate biomarkers, such as, complexes impregnation into the pores [26,27], encapsulation within the nanoparticle [28,29,30], immobilization by electrostatic interaction (cationic and anionic complexes) [31] or covalently-anchor onto the nanoparticle surface [12,32,33,34]. In this last case, the covalent bond avoids the complexes release in the cell environment and luminescence quenching compared to encapsulated architectures. The covalent bonding of the complexes on the silica surface is mediated by their previous modification with organosilanes that contain chelating groups (e.g., Schiff base, carboxylic acids, esters, ketones, pyridines), capable of binding to the first coordination sphere of the metal. In this sense, functionalization with a carboxylic acid, one of the least reported in the literature, draws attention due to the great versatility of this group, adding interesting biological properties to the material [35] such as increased biocompatibility, good colloidal stability [36], the possibility of conjugation with biomolecules [37] and an excellent chelating group for the coordination of several metals [38]. Accordingly, herein a step-by-step method, Scheme 1, is introduced to synthesize a hierarchical red-emitting hybrid based on covalent bonds of Eu^3+^-β-diketonates on the surface of decorated dense SiO_2_ nanoparticles assisted by monocarboxylic ligands featuring bioimaging application.

## 2. Materials and Methods

### 2.1. Materials

Tetraethylorthosilicate (TEOS) (Fluka, 99%), ammonium hydroxide (Synth, 24 26%), hydrochloric acid (Synth, 37%), methanol (Synth, 99,8%), ethanol (Synth, 99.5%), APTES (Synth, 98%), ninhydrin (Aldrich, 97%), chloroacetic acid (Sigma-Aldrich, 99.5%), potassium carbonate (Cinética, 99%), sodium hydroxide (Cinética, 97%), potassium methoxide (Aldrich, 97%), europium oxide (Aldrich, 99.99%) and Hdbm (Synth, 99.8%) were used without any further purification.

### 2.2. Synthesis of Silica Nanoparticles

For the synthesis of dense silica nanoparticles, the alkali-catalyzed sol-gel methodology (NH_4_OH) was used [39]. For this, in a beaker (100 mL), deionized water (5.59 mL), NH_4_OH (1.37 mL) and methanol (32.80 mL) were added. The mixture was kept under magnetic stirring at 25 °C (5 min); TEOS (3.60 mL) was slowly added and the solution was kept under stirring for 90 min. Sequentially, the stirring was stopped and the suspension rested for 12 h. The powder suspension was washed 3° times with methanol (12 mL) and centrifuged at 3800 rpm for 20 min. Finally, the sample was dried in a desiccator and afterward in an oven at 70 °C for 24 h. The silica sample synthesized will be hereafter labeled as S_1_. At the end of the process, 698.2 mg of S_1_ was obtained. Assignments for S_1_ Fourier Transform Infrared (FTIR) spectrum (KBr, cm^−1^): 3256 ν(O–H), 1630 δ(O–H, H_2_O), 1106 ν_as_(Si–O–Si), 949 ν(Si–O–H), 800 ν_s_(Si–O–Si), 470 δ_s_(Si–O–Si, dense network of ${\mathrm{SiO}}_{4}^{4-}$ tetrahedra) [40]. Attribution for S_1_^29^Si–NMR Q^n^ (ppm; %): Q^4^ (−111; 68.04), Q^3^ (−102; 27.99), Q^2^ (−93; 3.97).

### 2.3. Amino-Functionalization

In a beaker (100 mL), 0.5500 g of S_1_ was suspended in ethanol (35 mL) and kept in an ultrasonic bath (15 min). After that, APTES ethanolic solution was prepared by adding APTES (2.19 mL) in ethanol (15 mL). This solution was transferred to the beaker containing the S_1_ suspension under magnetic stirring and the silanization reaction proceeded vigorously at 25 °C (3 h). Then, the amino-functionalized nanoparticles were washed 3 times with ethanol (15 mL) and centrifuged at 3800 rpm (10 min). The powder was dried in a desiccator and then oven-dried at 70 °C (6 h), yielding a mass of 0.5210 g that hereafter will be named as S_1_N. Assignments for S_1_N FTIR spectrum (KBr, cm^−1^): 3243 ν(O–H), 1630 δ(O–H, H_2_O), 1545 δ(N–H), 1103 ν_as_(Si–O–Si), 951 ν(Si–O–H), 803 ν_s_(Si–O–Si), 471 δ_s_(Si–O–Si, dense network of ${\mathrm{SiO}}_{4}^{4-}$ tetrahedra) [40,41]. Attribution for S_1_N ^29^Si-NMR Q^n^ and T^n^ (ppm; %): Q^4^ (–111; 70.27), Q^3^ (–102; 24.05), Q^2^ (–93; 3.55), T^3^ (–67; 0.25), T^2^ (–60; 1.87).

### 2.4. Carboxyl-Functionalization

The carboxyl-functionalization was adapted from Reference [42], in which 0.2987 g of S_1_N—2.26 mmol of –NH_3_^+^ estimated by colorimetric method using ninhydrin [43]—was suspended in deionized water (20 mL) followed by the addition of K_2_CO_3_ (1.13 mmol) and left in ultrasound (15 min); after this time, the suspension was transferred to a round-bottom flask (50 mL). Thereafter, chloroacetic acid (4.52 mmol) was solubilized in deionized water (3 mL) and neutralized with K_2_CO_3_ (1.13 mmol); this solution was transferred to the reactional round-bottom flask. The reaction proceeded upon vigorous magnetic stirring at 60 °C (24 h). At the end of this time, the nanoparticles were washed twice with deionized water and twice with an aqueous solution of HCl (10^−3^ mol∙L^−1^). Finally, the sample was dried in a desiccator and then oven-dried at 70 °C, yielding 0.2449 g of the henceforth called S_1_NC sample. The carboxylic groups were quantified indirectly via the ninhydrin method [43]. Assignments for the S_1_NC FTIR spectrum (KBr, cm^−1^): 3452 ν(O–H), 2959 and 2928 ν_as_(C–H,CH_2_), 2855 ν_s_(C–H,CH_2_), 1743 ν(C = O), 1630 δ(O–H, H_2_O), 1105 ν_as_(Si–O–Si), 949 ν(Si–O–H), 800 ν_s_(Si–O–Si), 470 δ_s_(Si-O-Si, dense network of ${\mathrm{SiO}}_{4}^{4-}$ tetrahedra) [40].

After this step, the carboxylate-ligand salt (–COO^−^Na^+^) was prepared to promote the Eu^3+^ coordination. For that, the S_1_NC nanoparticles were suspended in water followed by the stoichiometric addition of NaOH (1 mmol∙L^−1^). The solvent was evaporated and the sample was oven-dried at 80 °C. Assignments for the salt S_1_NC FTIR spectrum (KBr, cm^−1^): 3259 ν(O–H), 1524 νas(COO^−^), 1414 ν_s_(COO^–^), 1630 δ(O–H, H_2_O), 1103 ν_as_(Si–O–Si), 949 ν(Si–O–H), 800 ν_s_(Si–O–Si), 471 δ_s_ (Si–O–Si, dense network of silicon tetrahedrons ${\mathrm{SiO}}_{4}^{4-}$) [40,44].

### 2.5. Coordination of Eu^3+^ to -COO^−^ Groups

In a round-bottom flask, 60 mg of S_1_NC—corresponding to 0.422 mmol of COO^−^ groups—was suspended with 10 mL of ethanol in ultrasound (5 min), then 0.464 mmol of Eu(NO_3_)_3_ in water (0.0455 mol∙L^−1^) was added. The reaction was kept under stirring (5 h) at 50 °C by using a reflux system. The sample was then washed 3 times with ethanol (15 mL) and centrifuged at 3800 rpm (10 min, 25 ºC). Finally, the powder was dried in a desiccator and in an oven (6 h), producing 61.1 mg of the hereafter labeled S_1_-[Eu] sample. Assignments for the S_1_-[Eu] FTIR spectrum (KBr, cm^−1^): 3415 ν(O–H), 2924 νas(C–H, CH_2_), 2852 ν_s_(C–H, CH_2_), 1524 ν_as_(COO^−^), 1414 ν_s_(COO^−^), 1635 δ(O–H, H_2_O), 1385 ν(${\mathrm{NO}}_{3}^{-}$), 1107 ν_as_(Si–O–Si), 951 ν(Si–O–H), 798 ν_s_(Si–O–Si), 467 δ_s_(Si–O–Si, dense network of ${\mathrm{SiO}}_{4}^{4-}$ tetrahedra) [40,45].

### 2.6. Displacement of Coordination Water Molecules by dbm^−^ Ligands

The synthesis methodology was adapted from Mutti [12]. In a beaker (50 mL), 40 mg of S_1_-[Eu] was transferred and suspended in ethanol (10 mL). The suspension was kept under an ultrasonic bath and then, it was transferred to a round-bottom flask (100 mL). The ligand Hdbm (195.5 mg, 0.87 mmol) was separately deprotonated with CH_3_OK (0.87 mmol in 10 mL of ethanol) and this solution was transferred dropwise to the reactional medium, that was kept under magnetic stirring at 50 °C (6 h). The suspension was washed 3 times with ethanol (15 mL) and centrifuged at 3800 rpm (10 min, 25 °C). Finally, the powder was dried in a desiccator and in an oven at 70 °C (6 h), leading to 45.5 mg of the sample, hereafter named S_1_-[Eu(dbm)]. Assignments for the S_1_-[Eu(dbm)] FTIR spectrum (KBr, cm^−1^): 3419 ν(O–H), 3060 ν(C–H, aromatic), 2959 and 2922 ν_as_(C–H, CH_2_), 2851 ν_s_(C–H, CH_2_), 1596 ν(C = O), 1548 ν_s_(C = C), 1522 ν_s_(C = O), 1478 and 1456 δ_as_(C–H, aromatic), 1222 and 602 δ(C–H, aromatic)_in plan_, 1099 ν_as_(Si–O–Si), 957 ν(Si–O–H), 799 ν_s_(Si–O–Si), 745 and 718 ν(C–H, aromatic), 683 δ(C–H, aromatic)_out plane_, 617 ν(Eu–O), 467 δ_s_(Si–O–Si, dense network of ${\mathrm{SiO}}_{4}^{4-}$ tetrahedra) [27,40].

### 2.7. Instrumentation

Images of samples were achieved from a Field Emission Gun Scanning Electron Microscopy (SEM FEG) JEOL model 7500F using an ethanolic suspension of nanoparticles dropped onto a Si substrate, sequentially coated with carbon by sputtering. Histograms were constructed by counting 100 nanoparticles using ImageJ (version 1.53e) [46]. FTIR spectra in KBr pellets were carried out in a Bruker model Tensor 27 spectrophotometer from 400–4000 cm^−1^ and increment of 4 cm^−1^. The –NH_2_ quantification was performed by using the ninhydrin colorimetric test [43] based on the absorption of Ruhemann’s purple compound using a Shimadzu model UV-1800 spectrophotometer (double beam) and ethanolic ninhydrin solution 5% (wt./v) as reference. Silicon Nuclear Magnetic Resonance (^29^Si {^1^H} CP/MAS NMR and ^29^Si MAS NMR) spectra were obtained on a Bruker Avance III HD 400 WB (9.4 T) spectrometer, with a 4 mm cross-polarization/magic-angle spinning (CP/MAS) probe and a maximum rotation frequency of 15 kHz, operating at temperatures between −140–150 °C. Thermogravimetry was carried out in a SDT-Q600TA equipment, from 25–1000 °C in Pt crucible, a heating rate of 10 °C/min upon circulating air atmosphere (100 mL/min). Zeta potential of the particles was measured in a Zetasizer Nano Series, model Nano-ZS, equipment from Malvern Instruments, in triplicate by using a suspension of the particles in phosphate buffer (0.01 mol/L, pH = 7.64). The excitation and emission spectra were measured in a Horiba Jobin Yvon, model Fluorolog-3 spectrofluorometer—continuous Xe lamp (450 W) with double excitation and emission monochromator and an R 928 Hamamatsu photomultiplier. Time-resolved spectroscopy was carried out in a phosphorimeter equipped with a Xe bulb (5 J/pulse). Fluorescence microscopy images of CHO-k1 cells were performed in a Nikon Confocal Microscope model C2/C2si, equipped with an inverted microscope system (Eclipse Ti–E) capable of obtaining fluorescence and confocal images using 405 and 561 nm lasers. The preparation protocol of glass slides for fluorescence microscopy analysis is descript in Note S1 of Supplementary Material.

## 3. Results and Discussion

### 3.1. Structure and Morphology

**Figure 1:**
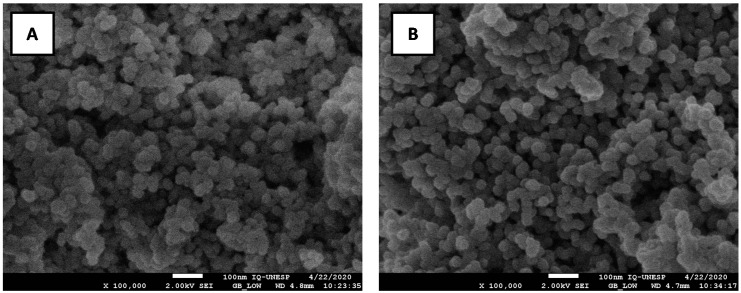
SEM FEG of (**A**) S_1_ and (**B**) S_1_-[Eu(dbm)].

SEM FEG images, Figure 1, confirm that spheroidal-shaped nanoparticles were obtained after the synthesis and that step-by-step process does not lead to any change of the particle size (average size of 32 ± 7 nm determined by Figure S1A,B), suggesting none leaching of the SiO_2_ surface and that the method is topotactic. The chemical mapping of the S_1_-[Eu(dbm)] hybrid surface evaluated by Energy-Dispersive Spectroscopy (EDS), Figure S1C–E, confirms that Eu^3+^ is evenly distributed throughout the surface and remains anchored even after successive washing and centrifugation processes.

The chemical bond formation of the S_1_-[Eu(dbm)] hybrid was elucidated by Fourier transform infrared (FTIR), Figure S2. S_1_-[Eu(dbm)] FTIR spectrum exhibits all the characteristic SiO_2_ vibrational modes. The anchoring of APTES on the SiO_2_ surface was confirmed by the vibrational mode at 1545 cm^−1^ related to the N-H bending of primary amines which vanishes in the S_1_NC sample due to the conversion of the N-H group into a secondary amine [40,41]. S_1_NC also shows the ν(C = O) stretch at 1743 cm^−1^ assigned to the COOH group and after the deprotonation, the vibrational modes at 1524 cm^−1^ and 1414 cm^−1^ were attributed to the antisymmetric (ν_as_) and symmetric (ν_s_) stretching vibrations of the COO^−^ group, respectively [40]. For the S_1_-[Eu] sample, those vibrational modes shift to 1506 cm^−1^ and 1419 cm^−1^ due to the coordination of Eu^3+^ to the COO^−^ group. The shift to lower wavenumbers (see Table S1) also ensures that Eu^3+^ coordinates itself to the COO^−^ group in a bidentate way according to the literature [44]. Furthermore, S_1_-[Eu] also displays a band at 1385 cm^−1^ characteristics of free ${\mathrm{NO}}_{3}^{-}$, probably as counter-ion and another one at 875 cm^−1^ assigned to ${\mathrm{NO}}_{3}^{-}$ coordinated to Eu^3+^ [47]. Finally, the Eu^3+^ coordination to dbm^−^ ligands was confirmed by the characteristic vibrational modes of the ligands (C-H stretching of aromatic rings at 3060 cm^−1^ and other vibrational modes of organic groups below 1600 cm^−1^) and the Eu-O stretching at 617 cm^−1^ [27].

**Figure 2:**
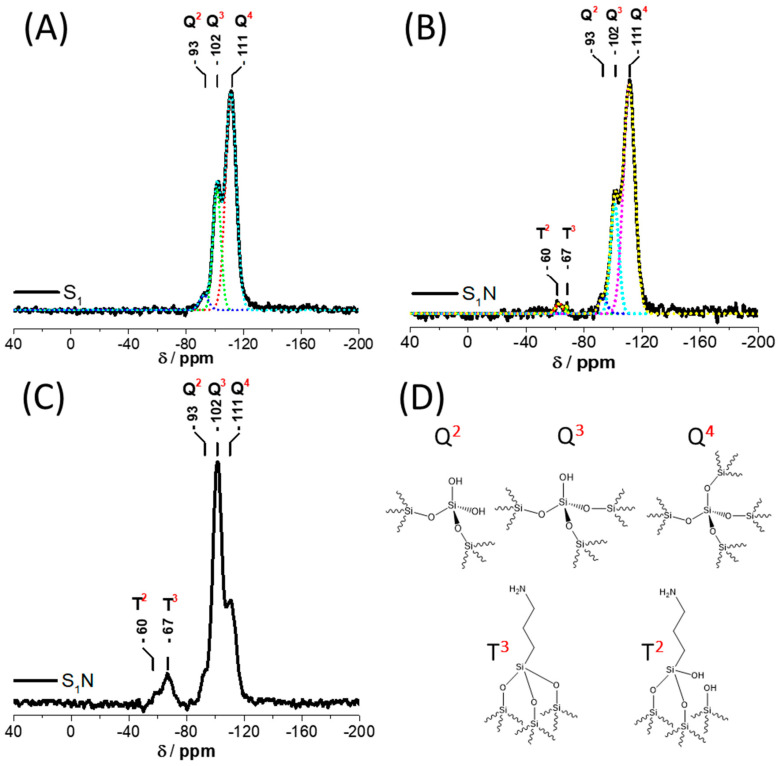
^29^Si MAS NMR spectra of (**A**) S_1_ and (**B**) S_1_N; the dashed lines represent deconvoluted curves obtained by using a Gaussian function. (**C**) ^29^Si {^1^H} CP/MAS NMR spectrum of S_1_N. (**D**) Illustration of Q^2, 3, 4^ and T^2, 3^ groups.

The covalent bond of APTES onto the SiO_2_ surface was investigated by using ^29^Si MAS NMR, Figure 2, where Q^4^ (−111 ppm), Q^3^ (−101 ppm) and Q^2^ (−93 ppm) groups were identified. The Q^4^ group is assigned to Si(O-Si)_4_ characteristic of the internal network of SiO_2_ particles, while Q^3^ and Q^2^ groups are associated with Si(O–Si)_3_(OH) and Si(O–Si)_2_(OH)_2_, respectively [30]. The APTES anchorage on the SiO_2_ surface was confirmed by the presence of T^2^ (−60 ppm) and T^3^ (−67 ppm) groups in the NMR spectrum, Figure 2B, which are characteristics of R–Si(O–Si)_2_(OH) and R-Si(O–Si)_3_, respectively, indicating that APTES bonds itself to the silica surface through bi- or tridentate modes, as illustrated in Figure 2D. From the area of the peaks in the 2^9^Si NMR spectra, the mol% of Q^n^ and T^n^ groups are determined before and after the APTES functionalization, Table 1. While the amount of Q^4^ groups represents around 68 mol% for S_1_, this amount increases up to 70 mol% for S_1_N. The amount of Q^2^ groups is almost invariant but the amount of Q^3^ decreases from ~28% to about 24%, for S_1_ and S_1_N, respectively. Since the Q^3^ group is bonded to one hydroxyl molecule, this variation indicates a decrease of 14% of the hydroxyl groups on the surface of the silica after the amino-functionalization. The amount of APTES molecules anchored onto the silica surface can be determined through the integration of T^n^ groups, that is 2.12 mol%; although it represents a small percentage, it is enough to enable the formation of complexes at the following steps, as indicated by FTIR and to obtain a highly emissive material as will be discussed later. To confirm the presence of T^n^ groups, it was performed the ^29^Si NMR at CP/MAS mode, which intensify signals associated with silicon atoms neighboring hydrogen atoms, Figure 2C.

The concentration of external amino groups (–NH_2_) was quantitatively estimated by the ninhydrin colorimetric assay described in the experimental procedure [43]. As a first visual test, S_1_N in ninhydrin solution becomes violet, confirming the presence of primary amines, Figure S3A. However, S_1_NC in ninhydrin solution displayed a yellowish-orange color, suggesting that the conversion of primary amines to secondary amines (not identified by ninhydrin) reached high yield. From the calibration curve using the Rheumann’s purple compound with a reliable absorbance at 578 nm, Figure S3B, the concentration of –NH_2_ groups was 7.58 mmol/g and 0.55 mmol/g for S_1_N and S_1_NC, respectively. From the difference between the concentration of –NH_2_ groups in both samples and assuming that –NH_2_ groups were converted to NH(CH_2_COOH), the concentration of carboxyl groups in the S_1_NC sample is estimated as 7.03 mmol/g—corresponding to 90% of conversion.

**Figure 3:**
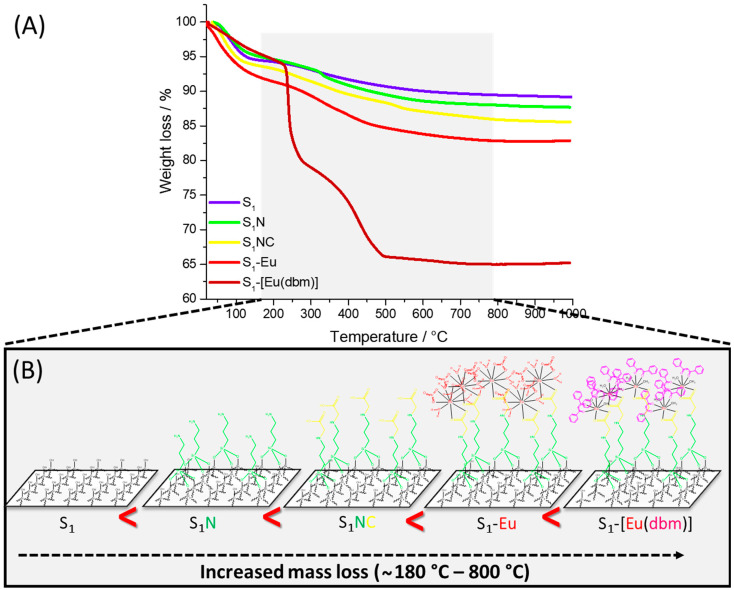
(**A**) Thermogravimetric curves of all samples obtained after each functionalization step. (**B**) Surface illustrative representation of all samples, elucidating the increase of organic matter losses during the second thermal event.

Modifications on the SiO_2_ nanoparticle surface was investigated by thermogravimetry. Two major thermal events are seen in Figure 3, the first that occurs around 200 °C is the release of water molecules adsorbed onto the SiO_2_ surface by hydrogen bondings involving silanol groups and the second event beginning at 200 °C is associated to the dehydroxylation silanol groups at the surface of the particles. After the functionalization processes, the combustion of organic matter anchored on the sample surface also contributes to the second thermal event. As expected, there is an increase in the weight loss of the second process, Table S2, due to the organic portion coming from the functionalization steps. The exact ending temperature of each process is indicated in Differential Thermogravimetry curves (DTG), Figure S4.

**Figure 4:**
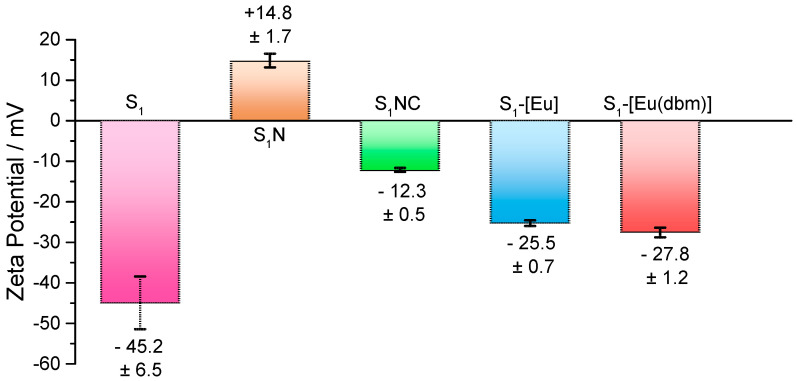
Zeta potential (pH = 7.64) for the particles synthesized in each functionalization step.

The surface charge of the hybrid in aqueous solution is a meaningful parameter considering its application as a biomarker and it was evaluated by zeta potential (ZP, at pH = 7.64, close to the physiological pH), Figure 4. For S_1_, at this pH value, the ZP is negative due to the deprotonation of silanol groups on the particle surface while for S_1_N, the –NH_2_ groups are protonated (–NH_3_^+^), giving a positive charge. After the carboxyl functionalization step, S_1_NC exhibits a negative charge due to the carboxyl group deprotonation, following the literature [48]. After the Eu^3+^ coordination to the carboxylate groups, ZP of S_1_-[Eu] is lowered due to the formation of negatively charged complexes containing ${\mathrm{NO}}_{3}^{-}$ ligands according to FTIR data. Finally, the dbm^−^ coordination does not lead to further changes in the ZP since dbm^−^ replaces ${\mathrm{NO}}_{3}^{-}$ ligands and both anions have the same charge.

### 3.2. Luminescence

**Figure 5:**
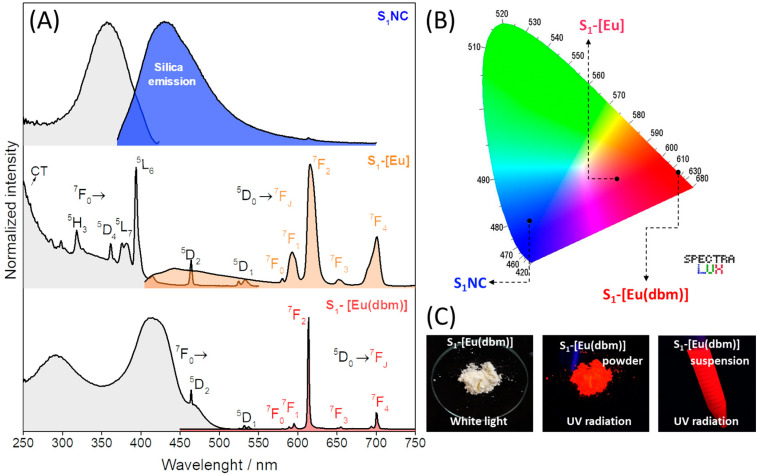
(**A**) Excitation and emission spectra (300 K) monitored at 433 and 356 nm for sample S_1_NC; 616 and 394 nm for sample S_1_-[Eu]; and 615 and 412 nm for sample S_1_-[Eu(dbm)], respectively. (**B**) 1931 CIE diagram of S_1_NC, S_1_-[Eu] S_1_-[Eu(dbm)] samples obtained from Reference [50]. (**C**) Photograph of S_1_-[Eu(dbm)] processed as powder or suspended in ethanol under white light and UV radiation (350 nm).

Considering the application of the hybrid as a biomarker, it is crucial to evaluate its luminescent features and for that, S_1_NC, S_1_-[Eu] and S_1_-[Eu(dbm)] had their excitation and emission spectra measured, as can be seen in Figure 5. S_1_NC displays broad excitation and emission bands peaking at 356 and 433 nm, respectively, which come from the SiO_2_ intrinsic luminescence. One explanation in literature for the SiO_2_ luminescence is related to the electron-hole recombination due to structural defects on the particle surface involving siloxane groups, generating defect-related electronic levels [49].

In the excitation spectrum of S_1_-[Eu], Figure 5A, the intraconfigurational *f–f* electronic transitions of Eu^3+^ are observed [51], while an intense broad absorption band bellows 300 nm is associated to O^2−^→Eu^3+^ charge transfer band (CT). Moreover, no excitation band associated with carboxylate groups is noticed, confirming its role in only anchoring Eu^3+^ to the SiO_2_ surface. Upon 394 nm excitation (Eu^3+ 7^F_0_→ ^5^L_6_ transition), the emission spectrum of S_1_-[Eu] is characterized by the typical ^5^D_0_→ ^7^F_0__–4_ Eu^3+^ electronic transitions within the red spectral region overlapped to the SiO_2_-related broad emission band in the blue spectral region. The quite broad profile of the Eu^3+^ emission bands is associated with different Eu^3+^ local microsymmetries on the particle surface [16]. In this sample the intensity is lower due to the presence of coordinated water molecules, which acts as a quenchers through multiphonon non-radiative processes due to O-H oscillators [52]. Similar results were found by Rocha [45] for Eu^3+^ incorporated into amorphous mesoporous SiO_2_.

To enhance the luminescence of S_1_-[Eu], dbm^-^ was coordinated to Eu^3+^ [27]. Its excitation spectra, Figure 5A, is dominated by broad and intense bands ranging 250–500 nm characteristic of dbm^−^ absorption, confirming that dbm^−^ sensitizes Eu^3+^ ion; some Eu^3+^ *f–f* absorption transitions with lower intensity are also observed. Interestingly, the dbm^−^ excitation bands fulfill the blue/violet excitation range, ensuring that the hybrid may be excited by using lower energetic wavelengths than the traditional energy within the near-UV spectral window used to excite downshift hybrids, which is, from the biological point of view, disadvantageous since UV radiation is dangerous to live organisms submitted to in vivo or in vitro assays [5]. The addition of dbm^−^ ligand leads to an enhancement of the intensity of the final S_1_-[Eu(dbm)] hybrid compared to S_1_-[Eu], making the Eu^3+^ emission bands narrower.

It is well-known that Eu^3+^ may act as a spectroscopic probe since some of its *f–f* transitions are sensitive to the ligand field around it, enabling further evaluations of Eu^3+^ local microsymmetry changes [53]. Specifically, the ratio between the emission band areas assigned to the forced electric dipole ^5^D_0_→^7^F_2_ transition and the magnetic dipole ^5^D_0_→^7^F_1_ transition enables evaluations of the asymmetry of the Eu^3+^ local sites. This ratio increases from 4.0–12.0 after the dbm^−^ coordination leading to a symmetry-lowering around Eu^3+^. This is an advantage since the ^5^D_0_→^7^F_2_ transition has its oscillator strength increased in low-symmetric sites [51].

The hybrid emission color was quantified by the colorimetric point of view by using the Commission Internationale de L’éclairage (CIE) 1931 diagram, Figure 5B. The (x,y) color coordinate of S_1_NC is (0.16, 0.16) within the blue spectral region with a dominant wavelength of 475 nm and color purity of 76%. After the Eu^3+^ coordination on the SiO_2_ surface, the color emission of S_1_-[Eu] moves towards the pink spectral region (0.47,0.29) due to the color mixture of Eu^3+^ and SiO_2_ emissions, with color purity of 29%. Finally, after the dbm^−^ coordination to Eu^3+^, the S_1_-[Eu(dbm)] emission color dislocates to the red spectral region (0.68, 0.31) achieving 100% of color purity and dominant wavelength of 615 nm, confirming that the dbm^−^ coordination was a valuable strategy to enhance the emission color purity and red light emission of the hybrid, as it can be seen by naked eyes, Figure 5C.

**Figure 6:**
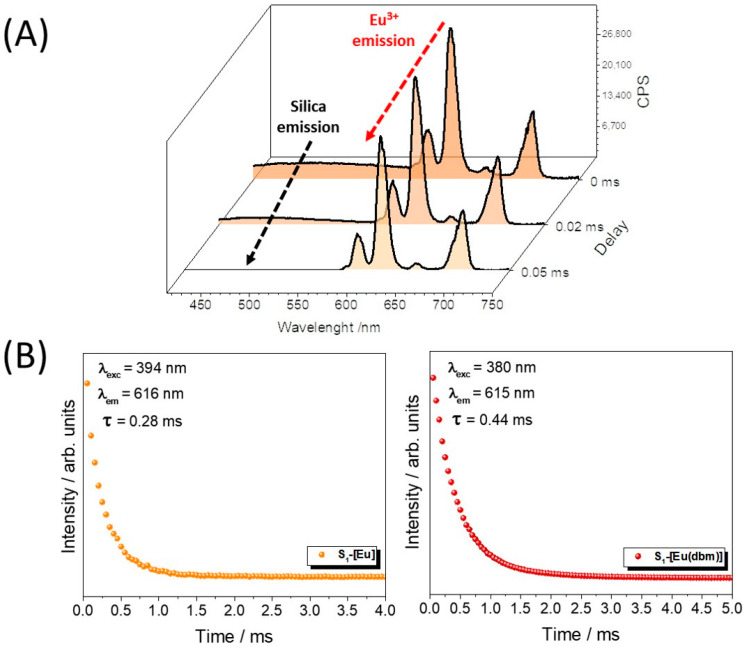
(**A**) Time-resolved emission spectra of S_1_-[Eu] with λ_ex_ = 394 nm; (**B**) ^5^D_0_ excited state decay curves of S_1_-[Eu] and S_1_-[Eu(dbm)].

To ensure that the Eu^3+^ emission can be differentiated from the SiO_2_ emission in S_1_-[Eu], time-resolved emission spectra were carried out, Figure 6A, confirming that a delay of 50 μs is enough to eliminate the SiO_2_ contribution to the emission spectrum. This delay time is close to the biological autofluorescence, demonstrating an advantage of using Ln^3+^-based biomarkers, for these later have a much longer emission lifetime allowing to distinguish between the biological emission from the hybrid emission [54]. On the other hand, for the final S_1_-[Eu(dbm)] hybrid, time-resolved spectroscopy is not necessary since the Eu^3+^ sensitization by dbm^−^ makes its emission much more intense than the SiO_2_ band even in steady-state acquisition mode, Figure 5A.

The photokinetic features of S_1_-[Eu] and S_1_-[Eu(dbm)] hybrids were further elucidated by emission decay curves, Figure 6B and the ^5^D_0_ excited state lifetimes (τ) are listed in Table 2. Since the Eu^3+^ ions can be inserted in several chemical environments when coordinated on the surface of the silica particle, we determined τ through the Inokuti–Hirayama model shown in Equation (1), which gives us a mean value of all excited-state lifetimes. As the ^5^D_0_ state lifetime depends on radiative (A_rad_) and nonradiative (A_nrad_) decay rates, τ = (A_rad_+A_nrad_)^−1^, it is possible to get the intrinsic emission quantum yield ($\phi_{Ln}^{Ln}$) of Eu^3+^—$\phi_{Ln}^{Ln}$ = A_rad_/(A_rad_ +A_nrad_) [55]. In this case, A_rad_ (A_rad_ = $\sum A_{0J}$) is determined from the emission spectrum by using Equation (2).

(1) $$\tau \;=\frac{\int_{0}^{\infty }I\left(t\right)t\;\mathrm{dt}}{\int_{0}^{\infty }I\left(t\right)\;\mathrm{dt}}$$

(2) $$A_{0\lambda }=A_{01}\left(\frac{S_{0j}}{S_{01}}\right)\left(\frac{\sigma_{01}}{\sigma_{0j}}\right),$$

where *I*(*t*) is the emission intensity at a time *t*, $A_{01}$ = (0.31 × $10^{-11}$) × $\eta^{3}$ × $\sigma_{0\to 1}^{3}$ and $\sigma_{0J\;}$ and $S_{0j}$ are the energy barycenter and the integrated intensity of the ^5^D_0_→ ^7^F_J_ transitions, respectively and η is the refractive index of SiO_2_ (1.435) [56].

Moreover, from τ values, it is possible to estimate the number of water molecules ($q_{H_{2}O}$) coordinated to Eu^3+^ by using the Horrocks and Sudnick model [57] (Equation (3)), where $\tau_{H_{2}O}$ is the ^5^D_0_ state lifetime of the hybrid in water suspension.

(3) $$q_{H_{2}O\;=\;}1.05\;\times \;\left[\frac{1}{\tau_{H_{2}O}}-\;\left(A_{01}\;\times \;\frac{\sum S_{0J}}{S_{01}}\right)\right].$$

After the dbm^−^ coordination to Eu^3+^ τ, A_rad_ and $\phi_{Ln}^{Ln}$ increases, in accordance with the Eu^3+^ sensitizing by the ligand. Moreover, the stoichiometric number of water molecules bonded to Eu^3+^ decreases from 3.5 to 1.6 after the dbm^−^ coordination, which agrees with the fact that dbm^−^ replaces H_2_O molecules. This leads to a decrease of the multiphonon quenching of the ^5^D_0_ stated, as it is indicated by the decrease of A_nrad_.

**Figure 7:**
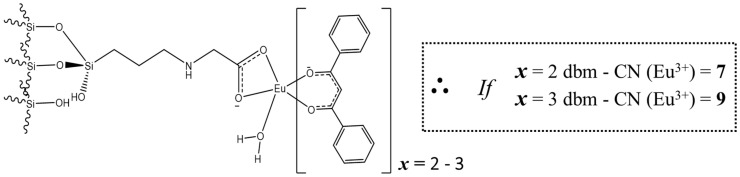
Eu^3+^ local sites on the SiO_2_ surface with coordination number (CN) seven or nine.

Emission-decay curves display multiexponential behavior, suggesting that there are more than one non-equivalent Eu^3+^ local sites, which agrees with the literature [12,58]. In this sense, by combining this information and the fact that Eu^3+^ is coordinated in a bidentate way to the carboxylate groups (as concluded by FTIR analysis), it is feasible to propose the structures shown in Figure 7 for Eu^3+^ local sites on the SiO_2_ particle surface. In those cases, the coordination number of Eu^3+^ is seven or nine.

By using $\phi_{Ln}^{Ln}$ as a figure of merit within the state-of-the-art of biomarkers fabricated by using covalently-bonded Ln^3+^-based hybrids, Table 3, the value reported by us is among the best and only few materials [59,60,61,62] feature higher values. Therefore, the finds reported here confirm that the step-by-step process was successful to synthesize violet/blue-to-red downshifting emitting S_1_-[Eu(dbm)] hybrids featuring high emission color purity and high intrinsic emission quantum yield, displaying potential for application as a luminescent biomarker.

To confirm this potentially, preliminary bioimaging tests were performed using fluorescence microscopy to investigate the interaction between the final hybrid S_1_-[Eu(dbm)] and CHO-k1 cells. Figure S5 shows the fluorescence microscopy images of CHO-k1 marked with DAPI blue-emitting nuclear stain and the red-emitting S_1_-[Eu(dbm)] hybrid. The tests were performed by exposing the cells to the hybrid for 2 h at concentrations of 62.5 and 125 μg/mL (for further details, see Note S1) [12,33]. The images in Figure S5 suggest that the hybrid was internalized by cells, crossing the cellular membrane and localized in intracellular regions at both concentrations tested. Besides, it was possible to confirm that the material keeps its luminescence in the red region even inside the cellular environment, suggesting that the medium does not cause changes in the hybrid structure and that it remains stable under physiological conditions. These preliminary characteristics corroborate that the material has potential for application in the field of bioimaging.

## 4. Conclusions

Herein, nanospheres of SiO_2_—average size of 32 nm—decorated with luminescent Eu^3+^-dbm complexes bonded by carboxylic bridges were successfully synthesized by a step-by-step method. The combination of thermogravimetry, FTIR, ^29^Si NMR and luminescence spectroscopy by using Eu^3+^ as a spectroscopic probe confirmed at least two non-equivalent Eu^3+^ local sites onto the SiO_2_ surface composed by Eu^3^+ bonded to two or three dbm^-^ ligands. The fabricated hybrid also matches (i) broad excitation band within the violet/blue spectral window, favoring the hybrid excitation using lower energy than the traditional UV applied in biological assays, (ii) intrinsic emission quantum yield (38%) among the best values reported so far for Eu^3+^-dbm based hybrids and (iii) intense red light emission featuring high emission color purity and relatively-long lifetime (0.44 ms). The results observed by fluorescence microscopy indicated that the hybrid was able to cross the cellular membrane and at the cytoplasm did not lose its red luminescence. Therefore, those finds confirm that this hybrid displays the potential to be applied as a biomarker for several imaging techniques such as time-resolved luminescence microscopy and confocal/fluorescence microscopy.

## Figures and Tables

**Table 1:** Percentage of groups obtained by deconvolution (Gaussian fitting) of ^29^Si NMR MAS spectra before and after the amino-functionalization process.

	S_1_ Sample	S_1_N Sample
Groups	Group Quantity in mol%	Group Quantity in mol%
**Q^4^**	68.04 ± 0.25	70.27 ± 0.40
**Q^3^**	27.99 ± 0.34	24.05 ± 0.63
**Q^2^**	3.97 ± 0.24	3.55 ± 0.43
**T^3^**	-	0.25 ± 0.06
**T^2^**	-	1.87 ± 0.17

**Table 2:** ^5^D_0_ excited state lifetime (τ), radiative (A_rad_) and nonradiative (A_nrad_) decay ratios, intrinsic emission quantum yield ($\phi_{Ln}^{Ln}$) and the number of coordinated H_2_O molecules ($q_{H_{2}O}$) for S_1_-[Eu] and S_1_-[Eu(dbm)].

Sample	τ/ms	A_rad_/s^−1^	A_nrad_/s^−1^	$\phi_{Ln}^{Ln}$	$q_{H_{2}O}$
**S_1_-[Eu]**	0.28	398	3225	11	3.5
**S_1_-[Eu(dbm)]**	0.44	864	1387	38	1.6

**Table 3:** State-of-the-art of luminescent hybrids containing dbm^−^ ligands coordinated to Eu^3+^ comparing $\phi_{Ln}^{Ln}$ as a figure of merit.

System	[ref]	Ligands	Host	$\phi_{Ln}^{Ln}$
Phen-Eu-DBM-SiO_2_/ZnO	[59]	dbm^−^ and phen	ZnO/SiO_2_	55.3
Eu–DBM–SiO_2_/ZnO	[59]	dbm^−^	ZnO/SiO_2_	22.6
Sil2N-EDTAEu-dbm	[60]	EDTA modified and dbm^−^	Silica Gel (commercial)	52.3
SiO_2_-APTES-cpa-Eu(DBM)_3_	[61]	dbm^−^	Mesoporous silica	41.1
Sample C	[62]	dbm^−^	ORMOSILs	39.2
Eu(DBMSi-SBA-15)_3_phen	[63]	dbm^−^ and phen	Mesoporous silica	32.7
bpy-Eu-DBM-S16	[64]	dbm^−^ and 2,2′-bipyridine	Mesoporous silica	7.7
SiO_2_-[Eu(SB)(dbm)_y_]	[12]	Schiff base and dbm^−^	Dense silica	32.0
Eu(DBM-SBA-15)_3_PMAA	[65]	dbm^−^ and PMAA^2^ polymer	Mesoporous silica	10.5
SiO_2_@Eu-DBM	[66]	dbm^−^	Mesoporous silica	14.0
Eu(ZnO-MAA-PHEMA-SBA-15)(DBM)_3_	[67]	dbm^−^ and silylated polymer	Mesoporous silica	22.3
Fe_3_O_4_|SiO_2_|1NEu	[68]	EDTA modified and dbm^−^	Fe_3_O_4_@SiO_2_	36.0
Eu(Dbm-Si)_3_ Phen/SiO_2_	[69]	dbm^−^ and phen	Silica	9.0
S_1_-[Eu(dbm)]	This study	dbm^−^ and carboxylic acid	Dense silica	38.4

phen = 1,10-phenanthroline; PMMA = poly(methyl methacrylate); PHEMA = poly 2-hydroxyethyl methacrylate; EDTA = ethylenediaminetetraacetic acid; ORMOSILs = Organically modified silicates.

## Supplementary Materials

The following are available online at https://www.mdpi.com/1996-1944/13/23/5494/s1, Figure S1. Histograms showing the diameter and standard deviation of the samples (A) S_1_ and (B) S_1_-[Eu(dbm)]. EDS spectra of (C) S_1_ e (D) S_1_-[Eu(dbm)]. (E) Surface chemical mapping of S_1_-[Eu(dbm)] suggesting a homogenous distribution of Si and Eu; Figure S2. FTIR spectra of all synthesized samples (left); magnification within the 1800–1300 cm^−1^ range (middle) and magnification within the 900–850 cm^−1^ range (right); Table S1. Position of the symmetric (ν_s_) and antisymmetric (ν_as_) stretching vibrations to determine the coordination modes of the carboxylate groups to Eu^3+^. Figure S3. (A) Qualitative test using ninhydrin to identify and compare the presence of primary amines in S_1_N and S_1_NC; (B) Calibration curve using APTES and ninhydrin. Figure S4. Thermogravimetric (TG) and first derivate (DTG) curves of all samples. Table S2. Weight loss assigned to the two thermal events obtained from the comparison of TG and DTG curves. **Note S1.** Slide preparation protocol for analysis by fluorescence microscopy. **Figure S5.** Fluorescence microscopy images of CHO-k1 rat cells cultivated in a suspension of 62.5 (top) and 125 µg/mL (bottom) of S_1_-[Eu(dbm)] and DAPI nuclear stain, using 40x of magnification and lens aperture of 0.95. (**A** and **D**) Excitation with laser 405 nm and read in the DAPI channel - blue; (**B** and **E**) excitation with laser 561 nm and read in the Texas Red channel - red; (**C**) overlapping images A and B; (**F**) overlapping images D and E.
