# Supplementary material for: Red-Emitting Hybrid Based on Eu3+-dbm Complex Anchored on Silica Nanoparticles Surface by Carboxylic Acid for Biomarker Application
Source: Materials (Basel). 2020 Dec 2;13(23):5494. doi: 10.3390/ma13235494 (PMC7731015; doi:10.3390/ma13235494)
Supplement: Supplementary file 1 [file materials-13-05494-s001.pdf]

materials

Supplementary Materials

Red-Emitting Hybrid Based on Eu^{3+}-dbm Complex Anchored on Silica Nanoparticles Surface by Carboxylic Acid for Biomarker Application

João A. O. Santos [1,2,3], Alessandra M. G. Mutti [1,2], Airton G. Bispo-Jr [3], Ana M. Pires [1,2,3] and Sergio A. M. Lima [1,*].

1 São Paulo State University (Unesp), School of Technology and Sciences, Presidente Prudente SP 19060-900, Brazil; joao.antonio@unesp.br (J.A.O.S.); alegarbosamutti@gmail.com (A.M.G.M.); ana.maria@unesp.br (A.M.P.)
2 São Paulo State University (Unesp), Institute of Biosciences, Humanities and Exact Sciences, São José do Rio Preto SP 15054-000, Brazil
3 São Paulo State University (Unesp), Institute of Chemistry, Araraquara SP 14800-900, Brazil; airton.germano.junior@gmail.com
* Correspondence: sergio.lima@unesp.br; Tel.: +55-18-3229-5752

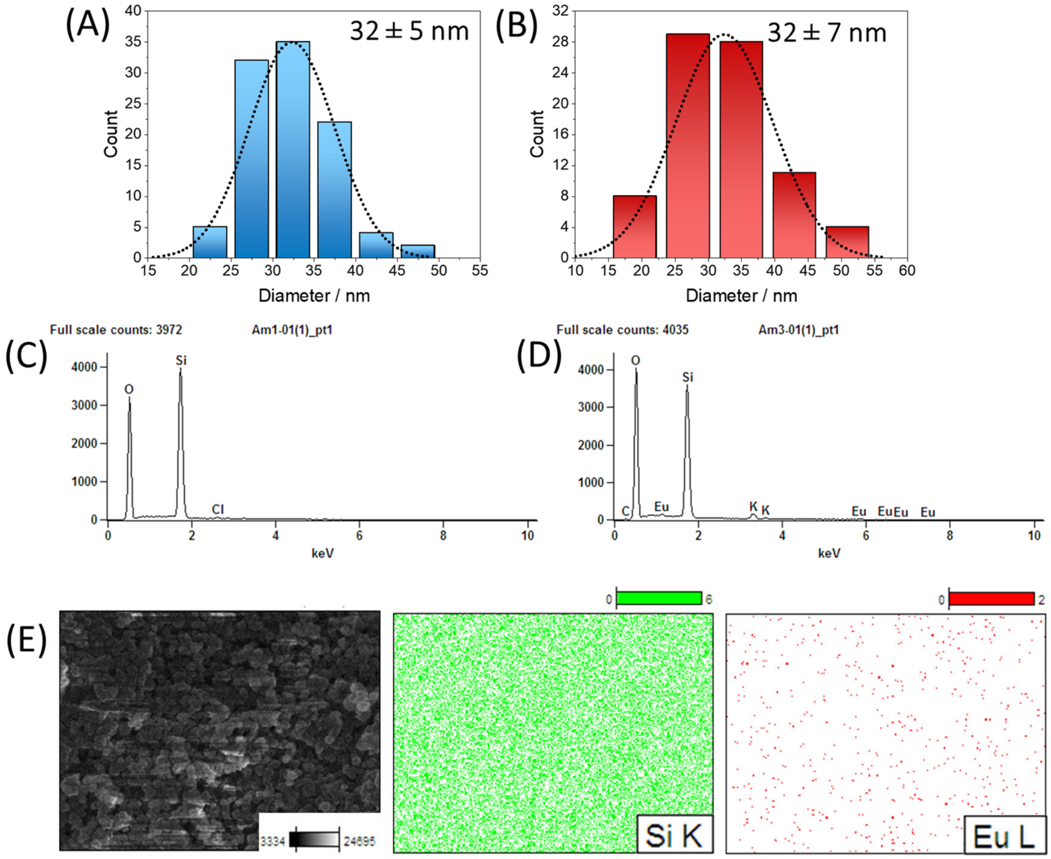

Figure S1. Histograms showing the diameter and standard deviation of the samples (**A**) S_1 and (**B**) S_1 [Eu(dbm)]. EDS spectra of (**C**) S_1 e (**D**) S_1-[Eu(dbm)]. (**E**) Surface chemical mapping of S_1-[Eu(dbm)] suggesting a homogenous distribution of Si and Eu.

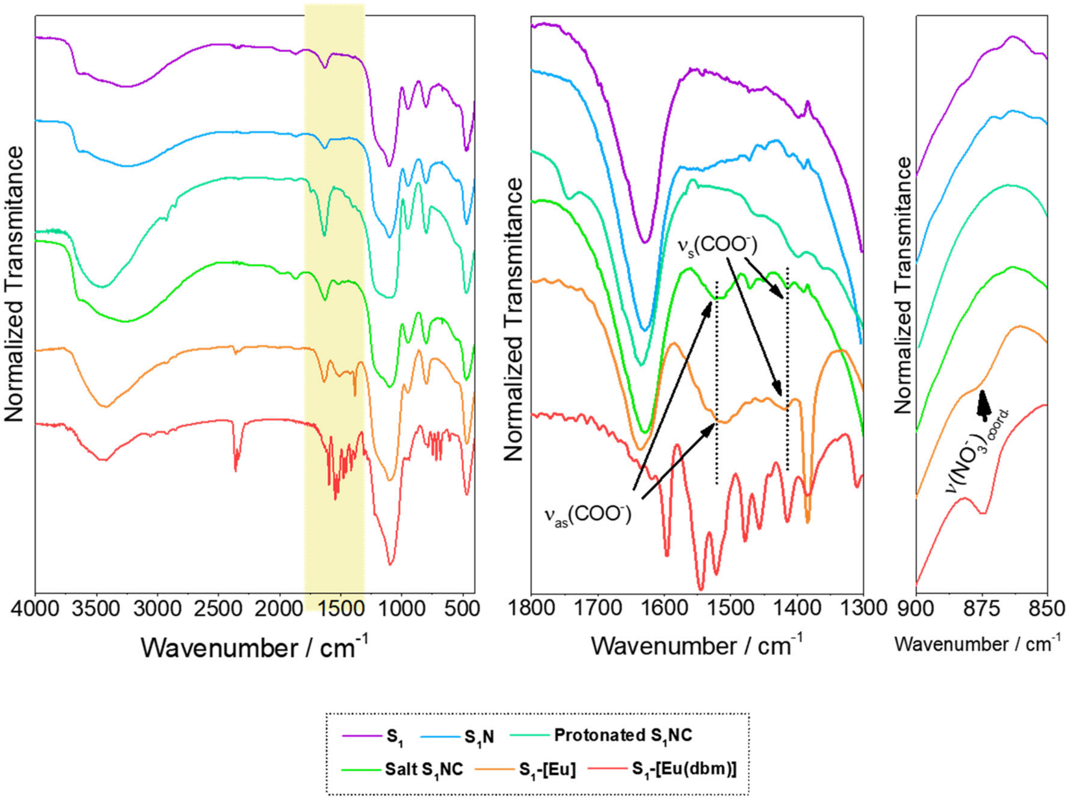

Figure S2. FTIR spectra of all synthesized samples (left); magnification within the 1800–1300 cm^{-1} range (middle) and magnification within the 900–850 cm^{-1} range (right).

Table S1. Position of the symmetric (ν_s) and antisymmetric (ν_{as}) stretching vibrations to determine the coordination modes of carboxylate groups to Eu^{3+}.

**Sodium Salt S_1NC**			**S_1-[Eu]**		
**ν_{as} / cm^{-1}**	**ν_s / cm^{-1}**	**$\Delta\nu$ / cm^{-1}**	**ν_{as} / cm^{-1}**	**ν_s / cm^{-1}**	**$\Delta\nu$ / cm^{-1}**
1,524	1,414	110	1,506	1419	87

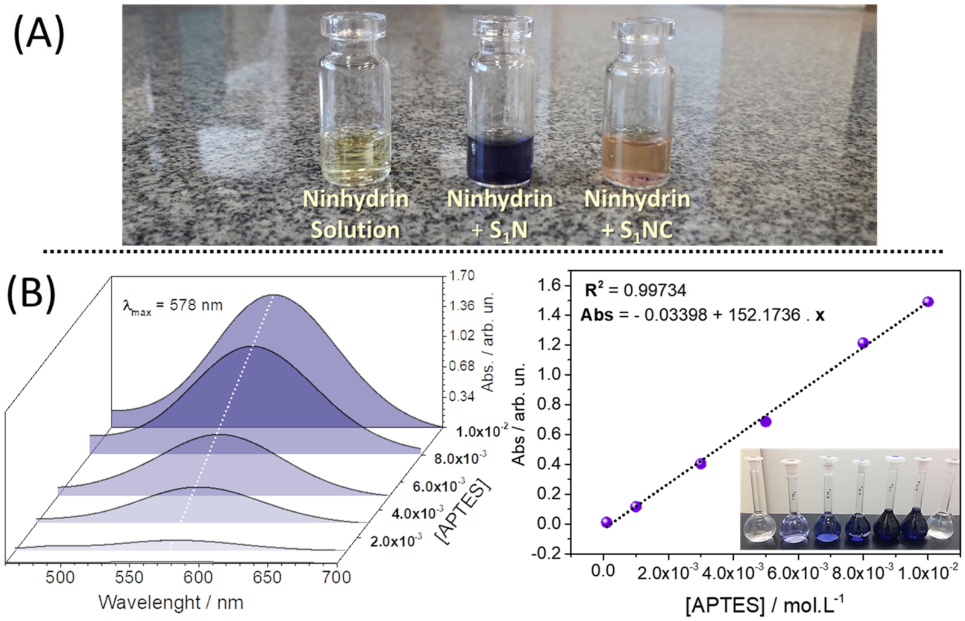

Figure S3. (**A**) Qualitative test using ninhydrin to identify and compare the presence of primary amines in S_1N and S_1NC; (**B**) Calibration curve using APTES and ninhydrin.

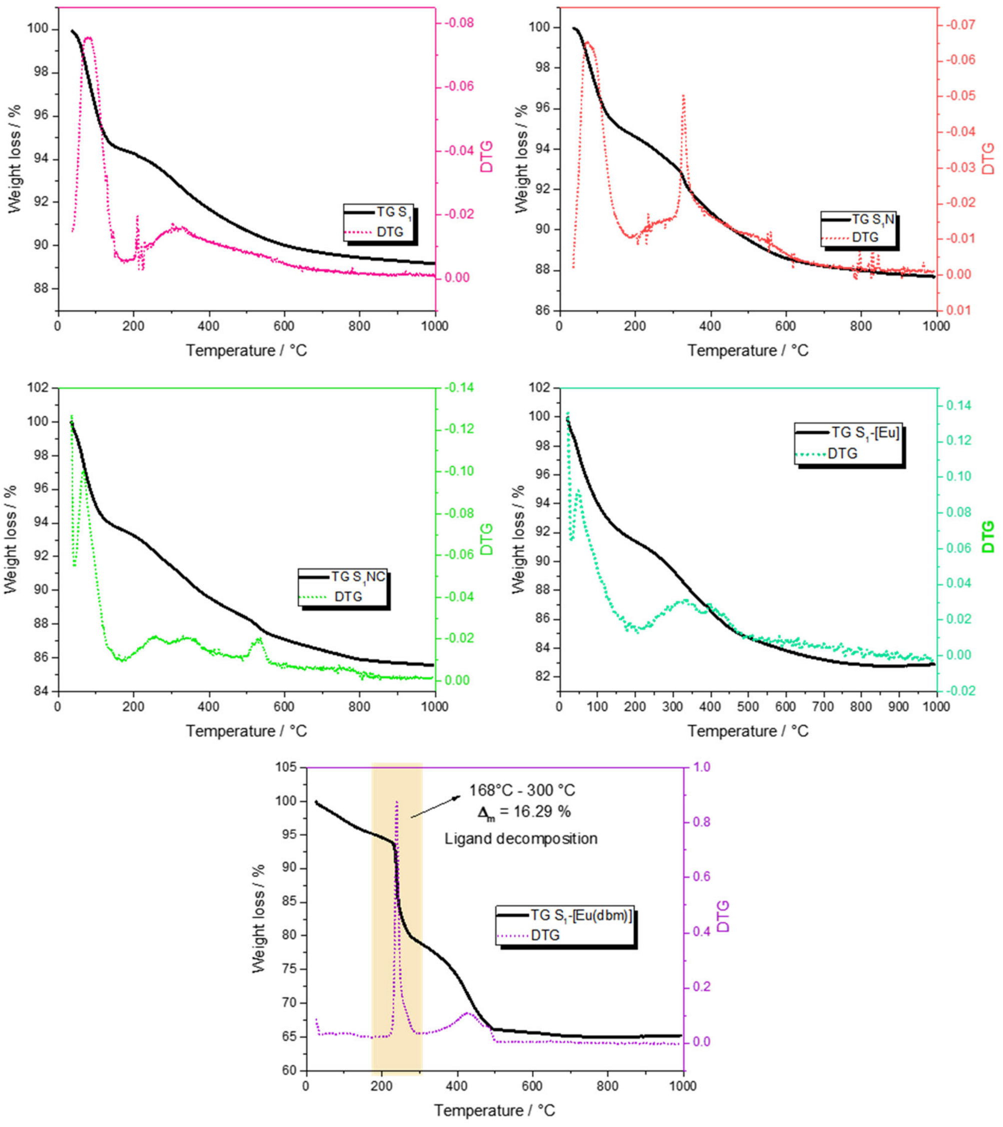

Figure S4. Thermogravimetric (TG) and first derivate (DTG) curves of all samples.

Table S2. Weight loss assigned to the two thermal events obtained from TG and DTG curves.

Sample	wt. % up to ~ 200 °C	wt. % ~ 200–800 °C
S_1	5.60	4.94
S_1N	5.26	6.85
S_1NC	6.39	7.71
S_1-[Eu]	8.61	8.56
S_1-[Eu(dbm)]	4.69	30.28

Supplementary Note: S1.

Slide preparation protocol for analysis by fluorescence 42 microscopy 43 To investigate the bioimaging capacity of the S1-[Eu(dbm)] hybrid by fluorescence microscopy, CHO-k1 cells 44 (adult

Chinese hamster ovary fibroblast cell line - cell culture was acquired from the Rio de Janeiro cell bank/BCRJ-0069) were incubated in a coverslip at a density of 6.95 x 105 cells per well in a culture dish for 24 h at 37 °C. The coverslips with the adhered cells were washed with PBS 0.1 mol.L-1 buffer and exposed to the nanoparticles of the final hybrid S1-[Eu(dbm)] in culture medium for a period of 2 hours. Then, they were washed three times with PBS, and marked with the nuclear dye DAPI (4',6-diamidino-2-phenylindol, dihydrochloride; Invitrogen, CA, USA; D1306) for 5 minutes. The blockade was performed with 3% BSA for 25 minutes. Finally, the coverslips were washed with PBS solution, fixed with formaldehyde (3.7% Aldrich), placed on a glass slide containing the mounting medium (50% glycerol in PBS), and inspected using a Confocal Laser Scanning Microscope at the fluorescence mode.

© 2020 by the authors. Submitted for possible open access publication under the terms and conditions of the Creative Commons Attribution (CC BY) license (http://creativecommons.org/licenses/by/4.0/).
